# A robust variable-structure LQI controller for under-actuated systems via flexible online adaptation of performance-index weights

**DOI:** 10.1371/journal.pone.0283079

**Published:** 2023-03-16

**Authors:** Omer Saleem, Jamshed Iqbal, Muhammad Shahzad Afzal

**Affiliations:** 1 Department of Electrical Engineering, National University of Computer and Emerging Sciences, Lahore, Pakistan; 2 School of Computer Science, Faculty of Science and Engineering, University of Hull, Hull, United Kingdom; 3 Department of Electrical and Software Engineering, University of Calgary, Calgary, Canada; National Institute of Technology, India (Institute of National Importance), INDIA

## Abstract

This article presents flexible online adaptation strategies for the performance-index weights to constitute a variable structure Linear-Quadratic-Integral (LQI) controller for an under-actuated rotary pendulum system. The proposed control procedure undertakes to improve the controller’s adaptability, allowing it to flexibly manipulate the control stiffness which aids in efficiently rejecting the bounded exogenous disturbances while preserving the system’s closed-loop stability and economizing the overall control energy expenditure. The proposed scheme is realized by augmenting the ubiquitous LQI controller with an innovative online weight adaptation law that adaptively modulates the state-weighting factors of the internal performance index. The weight adaptation law is formulated as a pre-calibrated function of dissipative terms, anti-dissipative terms, and model-reference tracking terms to achieve the desired flexibility in the controller design. The adjusted state weighting factors are used by the Riccati equation to yield the time-varying state-compensator gains.

## 1. Introduction

The idea of devising agile control procedures to regulate the behavior of under-actuated mechanical systems has garnered a lot of attraction among researchers owing to its immense applications in the fields of aircraft stabilization, marine-vessel stabilization, robotic manipulator tracking control, attitude control of satellites, and control of structural vibrations, etc [1]. By definition, under-actuated systems are identified as systems that possess a lesser number of control inputs as compared to the number of state-variables to be stabilized [2]. Having a lesser number of actuators is preferable because it minimizes the system’s energy expenditure, cost, and weight [3]. However, this configuration in conjunction with nonlinear system dynamics, complex coupling effects, and open-loop kinematic instability pose a complex control engineering problem [4]. Such systems demand a robust-optimal control law that can achieve the desired performance objectives even under the influence of exogenous disturbances [5].

### 1.1. Literature review

The Proportional-Integral-Derivative (PID) controller and its variants are simple to construct but they have the propensity to collapse under nonlinear disturbances [6]. The sliding-mode controllers offer a reliable and robust control yield; however, it comes at the cost of highly discontinuous control activity which unavoidably introduces chattering in the state response [7]. The ubiquitous neural and fuzzy control schemes are quite well-known for their flexibility to respond to and to reject bounded disturbances [8]. However, they rely upon empirically-defined rules or large sets of training data which are not only hard to acquire but also put an excessive computational expense on the embedded processor [9]. The Linear-Quadratic-Regulator (LQR) is a state-space controller that minimizes a quadratic performance index of the system’s state-variations and control-input to deliver the optimal control decisions [10]. Despite its benefits, the LQR is incapable to address identification errors, model variations, and environmental indeterminacies [11, 12]. The LQR can be retrofitted with auxiliary integral controllers to improve its robustness against uncertainties and load disturbances [13]. However, this augmentation slows down the system’s transient recovery behavior [14].

The aforementioned drawbacks of the LQR, and its variant(s), can be addressed by augmenting it with auxiliary online adaptation systems [12]. The dynamic adjustment of state-compensator gains offers a pragmatic approach to redesign the control law (after every sampling interval) to reject the transient disturbances in minimum time and attenuate the ensuing position-regulation fluctuations with minimal control energy expenditure while maintaining the controller’s stability throughout the operating regime [15]. The aforesaid objectives can be achieved via the state-dependent-Riccati-equation approach to formulate a nonlinear-quadratic-regulator for under-actuated systems. It uses well-identified State-Dependent-Coefficients (SDC) matrices to solve the Riccati equation, which eventually yields a time-varying state-feedback gain vector [16]. However, the nonlinear dynamics and complex geometry of the system make it very difficult to accurately identify the SDC matrices.

Recently, an innovative scheme to realize variable-structure (adaptive) LQRs has garnered a lot of attention [17]. This scheme is implemented by adaptively modulating the weighting matrices linked with the inner performance index. The state-weighting factors in the performance index are directly responsible for the manipulation of the corresponding state-variables [18]. Hence, the said scheme exploits this one-to-one correspondence by dynamically adjusting the state-weighting factors as a nonlinear function of state-error variables. Several well-postulated rule-based strategies that are formulated by pre-calibrated nonlinear hyperbolic scaling functions have thus been proposed in the scientific literature. E.g. the research reported in [19] strives to robustify the said variable-structure LQR design by proposing a flexible online weight-adjustment strategy that undertakes to increase the controller’s adaptability and Degree-Of-Freedom (DOF).

### 1.2. Proposed methodology

The novelty of the present research is to formulate a self-adaptive state-space controller for the under-actuated systems that use an innovative online adaptation mechanism for the weighting factors of the Quadratic-Performance-Index (QPI) to dynamically redesign the controller’s structure as the error conditions vary. The proposed control law employs a pre-calibrated Linear-Quadratic-Integral-Controller (LQIC) as the baseline controller that is retrofitted with the online adaptation law. The adaptation law uses state-error-dependent nonlinear functions to adjust the derivatives of the state weights. The derivatives are numerically integrated after every sampling interval. The updated weights are directly plugged into the Riccati equation for further computations that lead to online modification of the state-compensator gains. The main contributions of the present research work are as follows:

- Formulating a baseline adaptation scheme to adaptively modulate the state-weighting factors of the LQIC’s QPI via pre-calibrated nonlinear functions that are driven by dissipative terms and state-error-dependent anti-dissipative terms.
- Systematically restructuring the aforementioned nonlinear scaling functions to include auxiliary model-reference tracking terms in the baseline adaptation scheme as well.

The QNET rotary pendulum system is used as the benchmark platform to characterize and validate the performance of the proposed control scheme by conducting real-time hardware experiments [20].

The proposed adaptive control scheme offers several benefits. Firstly, the LQIC with fixed state-weighting factors cannot always deliver the best corrective action when the error conditions and system parameters are constantly changing over time. Hence, the proposed adaptive system obviates the necessity to affix the state-weighting factors offline which subsequently increases the controller’s adaptability to flexibly manipulate the control trajectory. Secondly, the adaptation scheme varies the state-weighting factors within the pre-defined limits which guarantee the asymptotic stability of the control law. This also prevents actuator saturation which may lead to wind-up or system collapse.

Thirdly, the proposed nonlinear adaptation law uses pre-calibrated dissipative terms, anti-dissipative terms, and model-reference tracking terms to improve the system’s flexibility to adaptively modulate the state-weighting factors. The acquisition of the information regarding the system as well as gain dynamics allows the controller to accurately realize the extent of degradation in the system’s time-domain response at any given instant. This knowledge, in turn, enables the adaptation law to demonstrate better self-reasoning, which subsequently leads to improved self-tuning of the weighting factors. The controller maintains a well-calibrated structure after every sampling instant, which simultaneously improves its response speed, damping against disturbances, and control efficiency. Finally, the proposed adaptation law can be easily programmed and solved using modern digital computers without putting an excessive recursive computational burden. The proposed variable structure LQIC design, using dissipative, anti-dissipative, and model-reference tracking terms to online adapt the state-weighting factors, has not been addressed earlier as per the knowledge of the authors. Hence, the key idea behind the research presented in this article is novel.

The remaining paper is organized as follows: The mathematical model and the baseline LQIC design for the RIP system is described in Section 2. The variable structure LQIC design is explained in Section 3. The two online adaptation schemes are systematically formulated in Section 4. The experimental results are presented and discussed in Section 5. Finally, the research is concluded in Section 6.

## 2. System description

**Fig 1:**
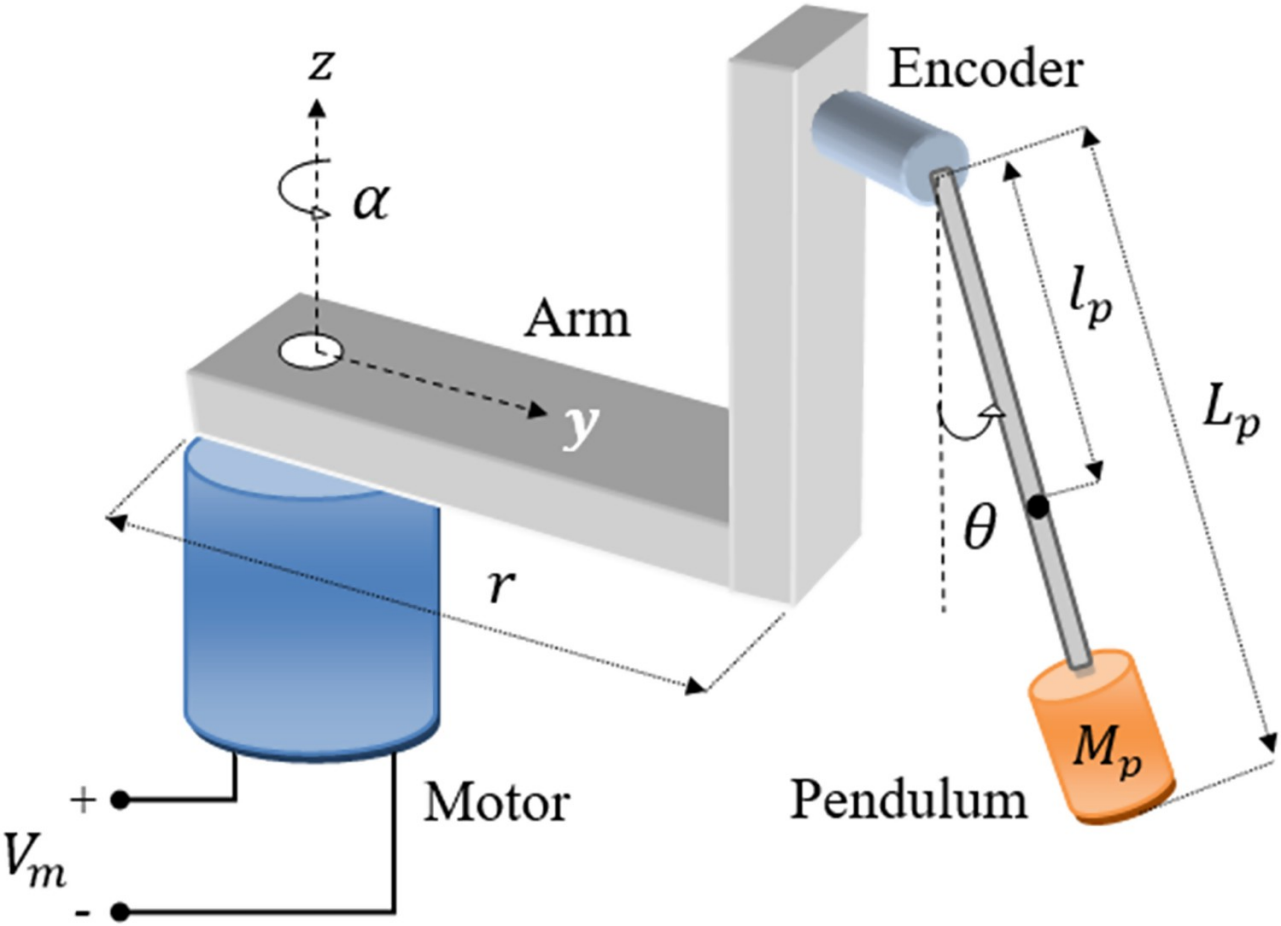
Schematic of a standard RIP system.

The Rotary-Inverted-Pendulum (RIP) system contains a vertical apparatus-rod connected to a horizontal rotating arm that is actuated by a DC-geared servo motor, as shown in Fig 1. The system requires a closed-loop feedback controller to stabilize the pendulum vertically while effectively tracking the reference position of the arm. The system’s model is derived using the Euler-Lagrange technique which uses the electrical and mechanical quantities involved in the system’s construction. The proposed feedback controller generates a variable input voltage signal to control the angular displacement of the DC motor. Correspondingly, the DC motor rotates the horizontal arm pivoted at its shaft. The arm rotations tend to displace the apparatus rod and provide the necessary energy to swing up and balance it vertically. As illustrated in Fig 1, the arm’s angular displacement is denoted as *α*, whereas the apparatus rod’s rotations about its pivot is denoted as *θ*. The aforesaid angular positions are acquired by dedicated rotary encoders that are commissioned with the motor’s shaft as well as the rod’s pivot respectively.

### 2.1. Mathematical model

The generalized angular position coordinates *α* and *θ* are used by the Lagrangian to model the system [21]. First of all, the system’s Lagrangian (*L*) is computed as the difference between the system’s total potential energy (*E*_*P*_) and potential energy (*E*_*P*_), as shown in (1).


$$L=E_{K}-E_{P}$$ (1)

$where,\;E_{P}=M_{p}l_{p}g(\cos\theta ),$

$and,\;E_{K}=\frac{1}{2}J_{e}{\left(\dot{\alpha }\right)}^{2}+\frac{1}{2}M_{p}{\left(r\dot{\alpha }-l_{p}\dot{\theta }(\cos\theta )\right)}^{2}+\frac{1}{2}M_{p}{\left(-l_{p}\dot{\theta }(\sin\theta )\right)}^{2}+\frac{1}{2}J_{p}{\left(\dot{\theta }\right)}^{2}$

The parameter details are mentioned in Table 1. The expression of the Lagrangian is computed as follows [22].


$$L=\frac{1}{2}(J_{e}+M_{p}r^{2}){\dot{\alpha }}^{2}+(\frac{2}{3}M_{p}{l_{p}}^{2}+\frac{1}{2}J_{p}){\dot{\theta }}^{2}-M_{p}l_{p}r\dot{\alpha }\dot{\theta }\;\cos\;\theta -M_{p}l_{p}g\;\cos\;\theta$$ (2)

**Table 1:** Model parameters of QNET rotary pendulum.

Parameter	Description	Identified value	Unit
*M*_*p*_	Mass of pendulum	0.027	kg
*l*_*p*_	Pendulum center of mass	0.153	m
*L*_*p*_	Length of pendulum rod	0.191	M
*r*	Length of horizontal arm	0.083	M
*M*_*arm*_	Mass of arm	0.028	Kg
*g*	Gravitational acceleration	9.810	m/s^2^
*J*_*e*_	Moment about motor shaft	1.23×10^−4^	kgm^2^
*J*_*p*_	Moment about pendulum	1.10×10^−4^	kgm^2^
*R*_*m*_	Motor armature resistance	3.30	Ω
*L*_*m*_	Motor armature inductance	47.0	mH
*K*_*t*_	Motor torque constant	0.028	Nm
*K*_*m*_	Back e.m.f. constant	0.028	V/(rad/s)
*T*_*m*_	Maximum torque	0.14	Nm

The nonlinear equations of motion are formulated using (3) [19].

$$\frac{\delta }{\delta t}(\frac{\delta L}{\delta \dot{\alpha }})-\frac{\delta L}{\delta \alpha }=\tau -b_{v}\dot{\alpha },\;\frac{\delta }{\delta t}(\frac{\delta L}{\delta \dot{\theta }})-\frac{\delta L}{\delta \theta }=0$$ (3)

where, *τ* is the DC motor control torque, and *b*_*v*_ represents the viscous friction in the DC motor, which is neglected in the model formulation owing to its negligible contribution. The DC motor torque is expressed as follows.


$$\tau =\frac{K_{t}(V_{m}-K_{m}\dot{\alpha })}{R_{m}}$$ (4)

The torque is a function of the DC motor’s input voltage *V*_*m*_. Using (4) in simplified form, the following set of nonlinear equation are obtained.


$$\ddot{\alpha }=\frac{-rM_{p}^{2}l_{p}^{2}g(\cos\theta )\theta -J_{p}M_{p}r^{2}\cos\theta \sin\theta {\left(\dot{\alpha }\right)}^{2}-(J_{p}+M_{p}l_{p}^{2})\tau }{(M_{p}r^{2}(\sin^{2}\theta )-J_{e}-M_{p}r^{2})J_{p}-M_{p}l_{p}^{2}J_{e}}$$ (5)

$$\ddot{\theta }=\frac{-M_{p}l_{p}((M_{p}r^{2}g(\sin^{2}\;\theta )-J_{e}g-M_{p}r^{2}g)\theta +rJ_{e}\;\sin\;\theta {\left(\dot{\alpha }\right)}^{2}-r\;\tau \;\cos\;\theta )}{(M_{p}r^{2}(\sin^{2}\theta )-J_{e}-M_{p}r^{2})J_{p}-M_{p}l_{p}^{2}J_{e}}$$ (6)

The system’s model is linearized about the vertical position; where, *α* = *π* rad., *θ* = 0, $\dot{\alpha }=0,\;\dot{\theta }=0$. The small angular displacements are dealt with by using the approximations; sin *θ*≈*θ* and cos *θ*≈1. These approximations yield the linearized state Eqs (7–8).


$$\ddot{\alpha }(t)=\frac{1}{H}\left(rM_{p}^{2}l_{p}^{2}g\theta (t)-\frac{(J_{p}+M_{p}l_{p}^{2})K_{t}K_{m}}{R_{m}}\dot{\alpha }(t)+\frac{(J_{p}+M_{p}l_{p}^{2})K_{t}}{R_{m}}V_{m}\right)$$ (7)

$$\ddot{\theta }(t)=\frac{1}{H}\left(M_{p}l_{p}g(J_{e}+M_{p}r^{2})\theta (t)-\frac{rM_{p}l_{p}K_{t}K_{m}}{R_{m}}\dot{\alpha }(t)+\frac{rM_{p}l_{p}K_{t}}{R_{m}}V_{m}\right)$$ (8)

$such\;that,\;H=J_{e}J_{p}+M_{p}r^{2}J_{p}+M_{p}l_{p}^{2}J_{e}$

The state-space representation of linear dynamical systems is expressed as (9).

$$\dot{x}(t)=Ax(t)+Bu(t),\;y(t)=Cx(t)+Du(t)$$ (9)

where, *x*(*t*) is the state-vector, *y*(*t*) is the output-vector, *u*(*t*) is the control input signal, ***A*** is the system matrix, ***B*** is the input matrix, ***C*** is the output matrix, and ***D*** is the feed-forward matrix. The system’s state-vector and input-vector are given in (10).


$$x(t)={\left[\alpha (t)\;\theta (t)\;\dot{\alpha }(t)\;\dot{\theta }(t)\right]}^{T},\;u(t)=V_{m}$$ (10)

The nominal linear state-space model of the RIP is given in (11) [20].


$$A=[\begin{array}{c} 0\;0\;1\;0\\ 0\;0\;0\;1\\ 0\;a_{1}\;a_{2}\;0\\ 0\;a_{3}\;a_{4}\;0 \end{array}],\;B=[\begin{array}{l} 0\\ 0\\ b_{1}\\ b_{2} \end{array}],\;C=[\begin{array}{c} 1\;0\;0\;0\\ 0\;1\;0\;0\\ 0\;0\;1\;0\\ 0\;0\;0\;1 \end{array}],\;D=[\begin{array}{l} 0\\ 0\\ 0\\ 0 \end{array}]$$ (11)

$$where,\;a_{1}=\frac{rM_{p}^{2}l_{p}^{2}g}{J_{p}J_{e}+J_{e}l_{p}^{2}M_{p}+J_{p}M_{p}r^{2}},\;a_{2}=\frac{-K_{t}K_{m}(J_{p}+M_{p}l_{p}^{2})}{(J_{p}J_{e}+J_{e}l_{p}^{2}M_{p}+J_{p}M_{p}r^{2})R_{m}},$$

$$a_{3}=\frac{M_{p}l_{p}g(J_{e}+M_{p}r^{2})}{J_{p}J_{e}+J_{e}l_{p}^{2}M_{p}+J_{p}M_{p}r^{2}},\;a_{4}=\frac{-rM_{p}l_{p}K_{t}K_{m}}{(J_{p}J_{e}+J_{e}l_{p}^{2}M_{p}+J_{p}M_{p}r^{2})R_{m}},$$

$$b_{1}=\frac{K_{t}(J_{p}+M_{p}l_{p}^{2})}{(J_{p}J_{e}+J_{e}l_{p}^{2}M_{p}+J_{p}M_{p}r^{2})R_{m}},\;b_{2}=\frac{rM_{p}l_{p}K_{t}}{(J_{p}J_{e}+J_{e}l_{p}^{2}M_{p}+J_{p}M_{p}r^{2})R_{m}}$$

The RIP’s model parameters are listed in Table 1 [23].

### 2.2. Linear quadratic integral controller design

The LQR uses the system’s linear state-space model and minimizes a QPI, expressed below, that considers the state and control input variations [24].

$$J_{lq}=\frac{1}{2}\int_{0}^{\infty }\left({x(t)}^{T}Qx(t)+{u(t)}^{T}Ru(t)\right)dt$$ (12)

where, ***Q***∈ℝ^4×4^ is a positive semi-definite state weighting matrix that penalizes the system state’s deviation from the equilibrium, and ***R***∈ℝ is a positive-definite input weighting matrix that penalizes the system’s control input. Here, ***Q*** and ***R*** matrices are denoted as follows.

$$Q=diag(q_{\alpha }^{*}\;q_{\theta }^{*}\;q_{\dot{\alpha }}^{*}\;q_{\dot{\theta }}^{*}),\;R=\rho$$ (13)

where, $q_{x}^{*}$ and *ρ* are real-numbered optimal coefficients of the ***Q*** and ***R*** matrices respectively. The allocation of a smaller *ρ* prompts the control law to apply unnecessarily large control energy under every operating condition, rendering it wasteful in such conditions. Similarly, a larger *ρ* yields insufficient control resources under every operating condition. Hence, to achieve a favorable balance between the system’s control economy and position-regulation capability, the value of *ρ* is selected as unity in this article. The state-compensator gains, acquired by using specific ***Q*** and ***R*** matrices, do not always guarantee an accurate reference-tracking and time-optimal behavior. Hence, in this research, the coefficient of ***Q*** matrix are tuned by minimizing the objective function (14) that considers the system’s classical state-error magnitude and its control-input energy [25].


$$J_{c}=\int_{0}^{\infty }\left({\left|e_{\alpha }(t)\right|}^{2}+{\left|e_{\theta }(t)\right|}^{2}+{\left|u(t)\right|}^{2}\right)dt$$ (14)

$such\;that,\;e_{\alpha }(t)=\alpha (0)-\alpha (t),\;e_{\theta }(t)=\pi -\theta (t)$

where, *e*_*α*_(*t*) and *e*_*θ*_(*t*) represent the position-regulation error of the arm and rod, respectively. The function *J*_*c*_ assigns equal weights to the control and state-error minimization criteria. The search space of the state-weighting factors is bounded within the limit [0, 100]. The tuning process begins with Q=diag(1111) and an exhaustive search is conducted in the direction of descending gradient of *J*_*c*_. In every trial, the pendulum is balanced for 5.0 seconds and the resulting cost is logged. The iterative tuning is terminated only when the minimum cost is achieved. The coefficients of ***Q*** and ***R*** matrices in this research are given in (15).


Q=diag(32.852.26.12.5),R=1 (15)

The attuned set of ***Q*** and ***R*** matrices is used by the matrix Riccati Eq (16) to evaluate the solution ***P*** offline.

$$A^{T}P+PA-PBR^{-1}B^{T}P+Q=0$$ (16)

where, ***P***∈ℝ^4×4^ is a symmetric positive-definite matrix. It is to be noted that the solution of matrix Riccati equation delivers an asymptotically stable control behavior as long as the weighting matrices are selected such that ***Q*** = ***Q***^*T*^≥0 and ***R*** = ***R***^*T*^>0. The state-compensator gain vector ***K*** is evaluated as,

$$K=R^{-1}B^{T}P$$ (17)

where, $K=[k_{\alpha }\;k_{\theta }\;k_{\dot{\alpha }}\;k_{\dot{\theta }}]$. The gain vector is computed as K=[−6.21130.56−4.2217.83]. The LQR law is expressed as,

u(t)=−Kx(t) (18)

The LQR law is also retrofitted with the state-error-integral variables given in (19).


$$\varepsilon_{\varphi }=\int_{0}^{t}e_{\varphi }(\tau )d\tau ,\;\varepsilon_{\theta }=\int_{0}^{t}e_{\theta }(\tau )d\tau$$ (19)

The integral control tend to improve the pendulum’s position-regulation accuracy and robustness against state fluctuations [26]. The integral control law is expressed as,

$$u_{i}(t)=K_{i}\varepsilon (t)=[K_{i\varphi }\;K_{i\theta }]\left[\begin{array}{c} \varepsilon_{\varphi }\\ \varepsilon_{\theta } \end{array}\right]$$ (20)

The integral-gain vector ***K***_*i*_ is optimized by minimizing the objective-function, *J*_*c*_, to damp the steady-state fluctuations. The integral gains are explored within the range [–5, 0]. The optimized integral gains are given as $K_{i}=[-2.06\;-7.47\times 10^{-6}]$. The modified linear control law expressed in (21), is formulated by linearly combining the conventional LQIC law with the aforementioned integral control law.


$$u(t)=-Kx(t)+K_{i}\varepsilon (t)$$ (21)

## 3. Variable structure LQIC design

The weighting factors are selected such that, *q*_*x*_≥0 and *ρ*>0. The rank of ***R*** is lesser than the system’s DOFs which validates the under-actuation property of the RIP system [17, 18]. Thus, it is hard to correlate and track the errors in all state variables using a single control input. However, the control-input dynamics can be used to manipulate the control-weighting factor. On the other hand, the state-weighting factors(*q*_*x*_) hold a one-to-one correspondence with the state variables. Hence, in this research, the control-weighting factor is fixed at unity while the state-weighting factors are chosen as the configurable objects. The state-weighting factors are dynamically adjusted using an online adjustment law to flexibly manipulate the control-input trajectory delivered by the LQIC. The online weight adjustment law is constituted via pre-calibrated nonlinear scaling functions that depends on the magnitude of classical state errors as well as their derivatives. The inclusion of aforesaid state-error variables in the weight adjustment law integrates the system DOFs into the weighting matrix, which helps in suppressing the detrimental effects of dynamics coupling. The time-varying state-weighting matrix is expressed as follows.

$$\bar{Q}=diag(sat(q_{\alpha }(t))\;sat(q_{\theta }(t))\;sat(q_{\dot{\alpha }}(t))\;sat(q_{\dot{\theta }}(t)))$$ (22)

where, *sat*(.) represents the saturation function of the following form.


$$sat(q_{x}(t))=\left\{ \begin{array}{l} (1+0.01M)q_{x}^{*},\;q_{x}(t)\geq (1+0.01M)q_{x}^{*}\\ q_{x}(t),\;(1-0.01P)q_{x}^{*}<q_{x}(t)<(1+0.01M)q_{x}^{*}\\ (1-0.01M)q_{x}^{*},\;q_{x}(t)\leq (1-0.01M)q_{x}^{*} \end{array}\right.$$ (23)

The saturation function is used to limit the unprecedented variations in the weighting-factors within ±*M*% of their nominal value $q_{x}^{*}$; wherein, $q_{\alpha }^{*}$ = 32.8, $q_{\theta }^{*}$ = 52.2, $q_{\dot{\alpha }}^{*}$ = 6.1, and $q_{\dot{\theta }}^{*}$ = 2.5. This restriction prevents the generation of discontinuous control activity and peak servo control signals which alleviates chattering and large state fluctuations in the response. Moreover, an unbounded enlargement in *q*_*x*_(*t*) leads to actuator saturation and wind-up; whereas, an unbounded reduction in it may eventually make *q*_*x*_(*t*)<0, which would render the control law unstable. In this research, the value of *P* is tuned by minimizing the objective function *J*_*c*_, and is thus set at *M* = 70.0. The updated $\bar{Q}$ matrix is used to solve the Riccati Eq, shown in (24), after every sampling interval to update the symmetric positive definite matrix $\bar{P}$.


$$A^{T}\bar{P}+\bar{P}A-\bar{P}BR^{-1}B^{T}\bar{P}+\bar{Q}=0$$ (24)

To maintain an economical control activity, the value of ***R*** is taken as unity. Finally, the modified matrix $\bar{P}$ is usedto dynamically adjust the state-compensator gain vector ***K***(*t*), as shown in (25).


$$K(t)=R^{-1}B^{T}\bar{P}$$ (25)

The adaptive optimal control law is redefined as shown in (26).


$$u(t)=-K(t)x(t)+K_{i}\varepsilon (t)$$ (26)

It is to be noted that the proposed weight-adjustment strategy only targets and adapts the state-weighting factors, which leads to the online adjustment of ***K***(*t*). Hence, the coefficients of ***K***_*i*_ are kept constant throughout at their prescribed values.

### Proof of stability

The closed-loop stability of the proposed adaptive control law is proved using the Lyapunov function shown in (27) [24].


$$V(t)={x(t)}^{T}P(t)x(t)>0,\;for\;x(t)\neq 0$$ (27)

The first derivative of this Lyapunov function is expressed as follows.


$$\dot{V}(t)=2{x(t)}^{T}P\dot{x}(t)$$ (28)

$$=2{x(t)}^{T}P(A-BK(t))x(t)$$

$$=2{x(t)}^{T}P(A-BR^{-1}B^{T}\bar{P})x(t)$$

$$={x(t)}^{T}(\bar{P}A+A^{T}\bar{P})x(t)-2{x(t)}^{T}(\bar{P}BR^{-1}B^{T}\bar{P})x(t)$$

By substituting Eq (24) in the above expression, the $\dot{V}(t)$ is simplified as shown in (29).


$$\dot{V}(t)=-{x(t)}^{T}\bar{Q}x(t)-{x(t)}^{T}(\bar{P}BR^{-1}B^{T}\bar{P})x(t)<0$$ (29)

The expression of $\dot{V}(t)$ is negative semi-definite as long as $\bar{Q}={\bar{Q}}^{T}\geq 0$ and ***R*** = ***R***^*T*^>0, which verifies the asymptotic convergence of the proposed controller. The online weight adaptation law is designed such that the coefficients of matrix $\bar{Q}$ are always kept positive semi-definite. The consequent (bounded) variations in $\bar{Q}$ are used to re-compute the Riccati Equation solution, after every sampling interval, which will yield a symmetric positive definite matrix $\bar{P}$ under every operating condition.

## 4. Online weight adaptation strategy

This section presents the constitution of the online weight adaptation strategy for the state-weighting factors. The arrangement is aimed at introducing flexible self-adaptability that enhances the response speed and strengthens the damping control effort against exogenous disturbances as well as intrinsic nonlinearities (such as, friction, backlash, cogging forces, air resistance, etc) while reducing the system’s large control input requirements. Two unique online weight-adaptation strategies have been investigated in this research.

### 4.1. Baseline weight-adjustment law

The LQI controller is augmented with a superior regulator that adaptively modulates the state-weighting factors as a function of state-error variations. The baseline weight-adaptation scheme used in this research is inspired by the Fisher’s gain-adjustment law due to its reasonable flexibility and good tracking capability [27]. The proposed adaptation scheme uses pre-configured dissipative and anti-dissipative functions. The weight-adjusting functions are formulated as first-order differential Eqs (30–33).

$$\dot{q_{\alpha }}(t)=-\sigma_{\alpha }q_{\alpha }(t)+\beta_{\alpha }e_{\alpha }^{2}(t)$$ (30)

$$\dot{q_{\theta }}(t)=-\sigma_{\theta }q_{\theta }(t)+\beta_{\theta }e_{\theta }^{2}(t)$$ (31)

$$\dot{q_{\dot{\alpha }}}(t)=-\sigma_{\dot{\alpha }}q_{\dot{\alpha }}(t)+\beta_{\dot{\alpha }}e_{\alpha }(t){\dot{e}}_{\alpha }(t)$$ (32)

$$\dot{q_{\dot{\theta }}}(t)=-\sigma_{\dot{\theta }}q_{\dot{\theta }}(t)+\beta_{\dot{\theta }}e_{\theta }(t){\dot{e}}_{\theta }(t)$$ (33)

where, *σ*_*x*_ and *β*_*x*_ respectively represent the predetermined positive decay rates and adaptation rates associated with each weight-adjusting function. These parameters are heuristically tuned offline by minimizing *J*_*c*_ to yield the best position-regulation behavior without imposing large control input requirements. The search spaces of *σ*_*x*_ and *β*_*x*_ are bounded within the limits [0, 1] and [0, 10], respectively. The tuning process begins at random values of these parameters and an exhaustive search is conducted in the direction of descending gradient of *J*_*c*_. In every trial, the pendulum is balanced for 5.0 seconds and the resulting cost is logged. The iterative tuning is terminated only when the minimum cost is achieved. The tuned values are *σ*_*α*_ = 0.016, *σ*_*θ*_ = 0.025, $\sigma_{\dot{\alpha }}$ = 0.010, $\sigma_{\dot{\theta }}$ = 0.018, *β*_*α*_ = 0.46, *β*_*θ*_ = 0.61, $\beta_{\dot{\alpha }}$ = 6.05, and $\beta_{\dot{\theta }}$ = 8.18. Each function is composed of the following dissipative and an anti-dissipative term.

$$\mathrm{Dissipative}\;\mathrm{term}\;\{\begin{array}{l} -\sigma_{\alpha }q_{\alpha }(t)\\ -\sigma_{\theta }q_{\theta }(t)\\ -\sigma_{\dot{\alpha }}q_{\dot{\alpha }}(t)\\ -\sigma_{\dot{\theta }}q_{\dot{\theta }}(t) \end{array}\;\mathrm{Anti}‐\mathrm{dissipative}\;\mathrm{term}\;\{\begin{array}{c} \beta_{\alpha }e_{\alpha }^{2}(t)\\ \beta_{\theta }e_{\theta }^{2}(t)\\ \beta_{\dot{\alpha }}e_{\alpha }(t){\dot{e}}_{\alpha }(t)\\ \beta_{\dot{\theta }}e_{\theta }(t){\dot{e}}_{\theta }(t) \end{array}$$

The contribution of the dissipative and anti-dissipative terms is described below:

1. The anti-dissipative term increases the rate-of-change of the proportional state-weighting factors (*q*_*α*_(*t*) and *q*_*θ*_(*t*)) as the magnitude of state errors increases.
2. The anti-dissipative term increases the rate-of-change of the differential state-weighting factors ($q_{\dot{\alpha }}(t)$ and $q_{\dot{\theta }}(t)$) as the response diverges from the reference position, and vice versa.
3. The dissipative term ‘exponentially’ reduces the rate-of-change of each state-weighting factor when the system is either approaching (and settling at) the reference position or when the anti-dissipative term is small.

The rationale described above dynamically modifies the state-weighting factors by considering their rate of inflation or depression. This arrangement dynamically redesigns the control law after every sampling interval, which yields a tight control effort to quickly realize and compensate for the exogenous disturbances and a soft control effort to improve position-regulation accuracy in the vertical (dynamic) equilibrium state [28]. This arrangement significantly increases the controller’s self-reasoning capability thus subsequently ensuring flexible manipulation of the applied control stiffness across the entire range of operating conditions.

**Fig 2:**
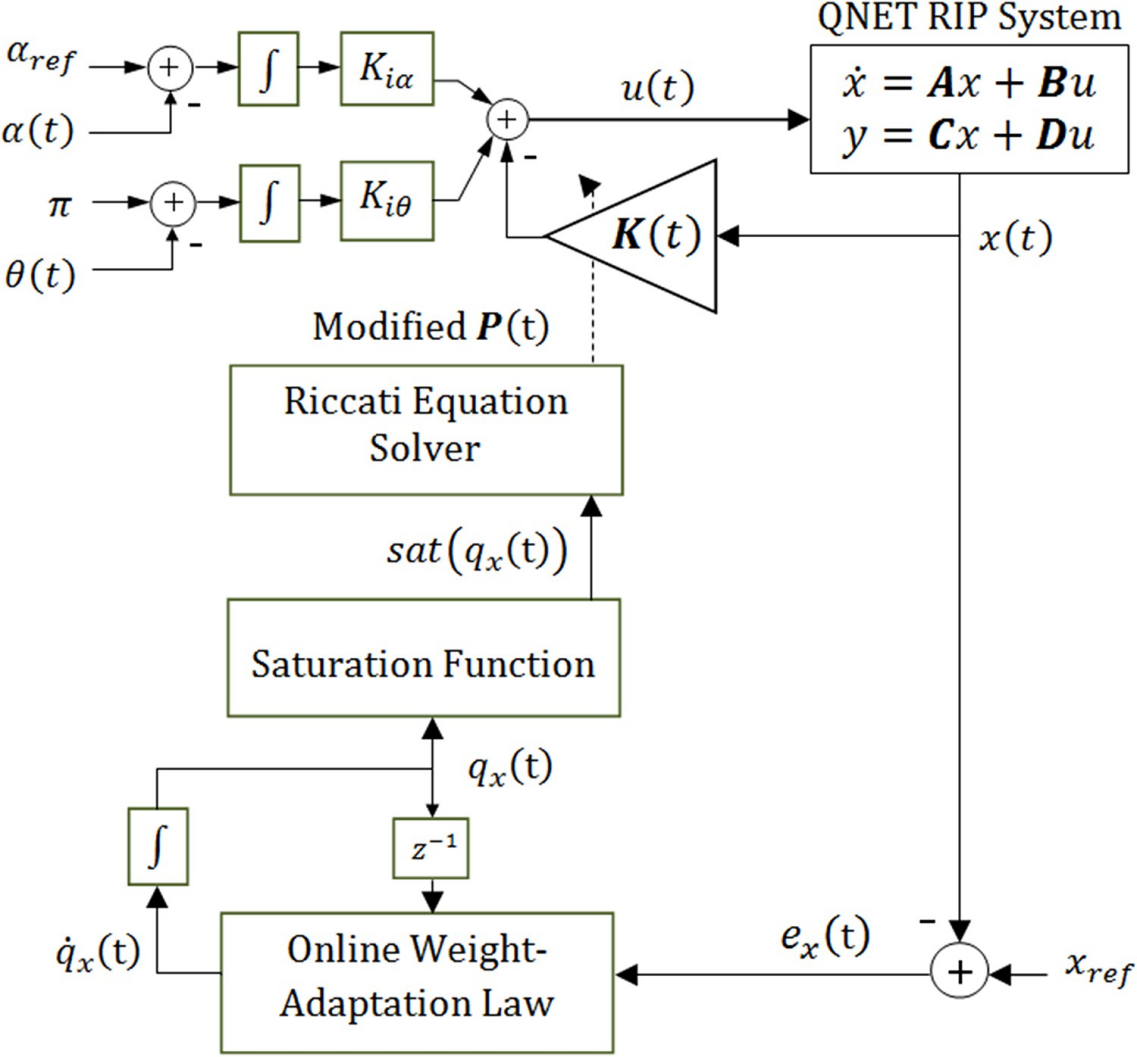
Baseline variable–structure LQIC procedure.

The aforesaid scheme does not require any prior knowledge of the system’s geometry. The weight adjustment is initiated from the preset values of the state-weighting factors; such that, $q_{x}(0)=q_{x}^{*}$. The weighting factors are updated once after every sampling instant by solving the first-order differential equation as described in (34). Consider the following general expression representing the aforesaid weight-adjusting function.

$$\dot{q_{x}}(t)=-\sigma_{x}q_{x}(t)+\beta_{x}z(t)$$ (34)

where, *z*(*t*) is the error-dependent function $e_{\alpha }^{2}(t),\;e_{\theta }^{2}(t)$, or $e_{\alpha }(t){\dot{e}}_{\alpha }(t)$. The solution of this first-order differential equation is computed as shown in (35).

$$q_{x}(t)=exp(-\sigma_{x}t)q_{x}(0)+\int_{0}^{t}\left(exp(-\sigma_{x}(t-p))\beta_{x}z(p)\right)dp$$ (35)

where *exp*(.) represents the exponential function. These computations can be easily handled by modern digital computers without putting an excessive recursive expense on them. After every sampling interval, the adjusted values of *q*_*x*_ are fed to the saturation function given in (23), to limit the variations within ±70.0% of the nominal value. This is done to comply with the stability requirements of LQI controller expressed in (29). The resulting saturated weights *sat*(*q*_*x*_(*t*)) are used to re-compute the solution of Riccati equation which serves to modify the state-compensator gains online. This control procedure is referred as Baseline-Variable-Structure LQIC (BVS-LQIC) in this article. The block diagram of the proposed BVS-LQIC procedure is shown in Fig 2.

### 4.2. Improved weight-adjustment law

**Fig 3:**
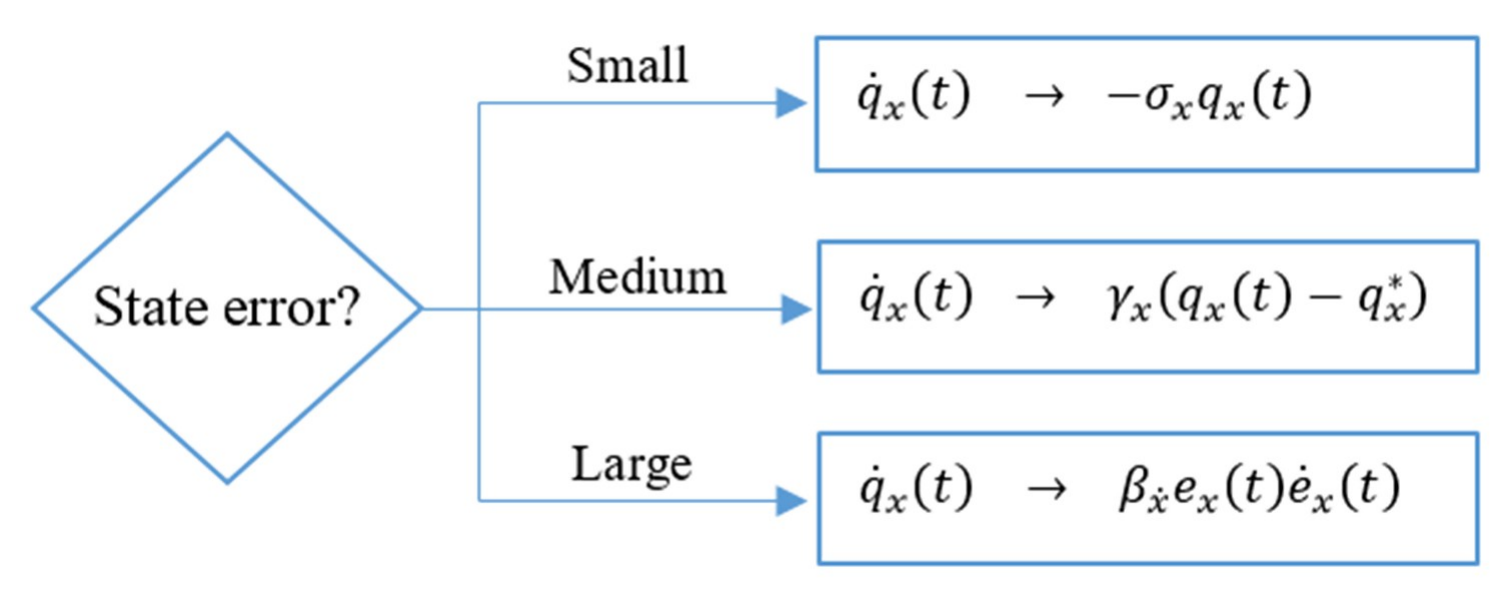
Flow chart for the improved weight–adjustment law.

The aforementioned online adaptation law is augmented with auxiliary components to further increase its flexibility and DOF [28]. This permits the adaptation strategy to improve the adaptability, self-learning, and self-regulation capability of the closed-loop control system. The said modification is incorporated by retrofitting the baseline adaptation law with an additional model-reference tracking term of the form $\gamma_{x}(q_{x}(t)-q_{x}^{*})$, apart from the already existing dissipative and anti-dissipative term. Under medium state error conditions, the adaptation law attempts to imitate the nominal control law expressed in (18), with weights $q_{x}^{*}$ to apply a mild control effort to avoid peak servo demands, prevent chattering, and suppress post-disturbance oscillations or overshoots. The inclusion of the model-reference tracking error regulator in the adaptation law allows the controller to precisely realize the extent of disturbance in the system at any given instant and then efficiently apply the necessary control action to compensate the bounded disturbances. The weight-adjustment law is synthesized as per the flow chart depicted in Fig 3.

The adaptation law traverses among the aforementioned phases of the state-error profile by employing a state-error-driven hyperbolic scant function *μ*_*S*,*x*_(*e*_*x*_), which approaches unity under small error conditions and zero under large error conditions. The hyperbolic secant functions formulated in (36), are used to apply weight and alter the contribution of each term in the adaptation law with the variation in error conditions.

$$\mu_{S,\alpha }(e_{\alpha })=\mathrm{sech}\left(\omega_{\alpha }e_{\alpha }\right),\;\mu_{S,\theta }(e_{\alpha })=\mathrm{sech}\left(\omega_{\theta }e_{\theta }\right),$$

$$\mu_{L,\alpha }(e_{\alpha })=1-\mu_{S,\alpha }(e_{\alpha }),\;\mu_{L,\theta }(e_{\theta })=1-\mu_{S,\theta }(e_{\theta }),$$

$$\mu_{M,\alpha }(e_{\alpha })=\mu_{S,\alpha }(e_{\alpha })\times \mu_{L,\alpha }(e_{\alpha }),\;\mu_{M,\theta }(e_{\theta })=\mu_{S,\theta }(e_{\theta })\times \mu_{L,\theta }(e_{\theta }).$$ (36)

where, *μ*_*S*,*x*_(.) steers the adaptation law under small error conditions, *μ*_*L*,*x*_(.) governs the law under large error conditions, and *μ*_*M*,*x*_(.) drives the law under medium error conditions. The parameter *ω*_*x*_ represents the variance of the hyperbolic secant function sech(.). To comply with the aforementioned rules, the dissipative terms are weighted with *μ*_*S*,*x*_(*e*_*x*_), the anti-dissipative terms are weighted with *μ*_*L*,*x*_(*e*_*x*_), and the model-referencetracking terms are weighted with *μ*_*M*,*x*_(*e*_*x*_). The modified weight-adjustment functions are expressed in (37–40).


$$\dot{q_{\alpha }}(t)=\frac{1}{1+\mu_{M,\alpha }(e_{\alpha })}\left(\beta_{\alpha }\mu_{L,\alpha }(e_{\alpha })e_{\alpha }^{2}(t)-\gamma_{\alpha }\mu_{M,\alpha }(e_{\alpha })(q_{\alpha }(t)-q_{\alpha }^{*})-\sigma_{\alpha }\mu_{S,\alpha }(e_{\alpha })q_{\alpha }(t)\right)$$ (37)

$$\dot{q_{\theta }}(t)=\frac{1}{1+\mu_{M,\theta }(e_{\theta })}\left(\beta_{\theta }\mu_{L,\theta }(e_{\theta })e_{\theta }^{2}(t)-\gamma_{\theta }\mu_{M,\theta }(e_{\theta })(q_{\theta }(t)-q_{\theta }^{*})-\sigma_{\theta }\mu_{S,\theta }(e_{\theta })q_{\theta }(t)\right)$$ (38)

$$\dot{q_{\dot{\alpha }}}(t)=\frac{1}{1+\mu_{M,\alpha }(e_{\alpha })}\left(\beta_{\dot{\alpha }}\mu_{L,\alpha }(e_{\alpha })e_{\alpha }(t){\dot{e}}_{\alpha }(t)-\gamma_{\dot{\alpha }}\mu_{M,\alpha }(e_{\alpha })(q_{\dot{\alpha }}(t)-q_{\dot{\alpha }}^{*})-\sigma_{\dot{\alpha }}\mu_{S,\alpha }(e_{\alpha })q_{\dot{\alpha }}(t)\right)$$ (39)

$$\dot{q_{\dot{\theta }}}(t)=\frac{1}{1+\mu_{M,\theta }(e_{\theta })}\left(\beta_{\dot{\theta }}\mu_{L,\theta }(e_{\theta })e_{\theta }(t){\dot{e}}_{\theta }(t)-\gamma_{\dot{\theta }}\mu_{M,\theta }(e_{\theta })(q_{\dot{\theta }}(t)-q_{\dot{\theta }}^{*})-\sigma_{\dot{\theta }}\mu_{S,\theta }(e_{\theta })q_{\dot{\theta }}(t)\right)$$ (40)

The parameters *β*_*x*_ and *σ*_*x*_ represent the adaptation rates and decay rates, respectively. Their values are prescribed in the sub-section 5.1. The nominal (reference) weights $q_{x}^{*}$ have also been prescribed already in Section 4. The parameter *γ*_*x*_ represents the predefined positive model-reference tracking rates associated with each function. The parameters *γ*_*x*_ and *ω*_*x*_ are empirically tuned offline by minimizing *J*_*c*_ to optimize the RIP’s position-regulation behavior and disturbance-compensation capability. The search spaces of *γ*_*x*_ and *ω*_*x*_ are restricted within the limits [0, 1]. The tuning process begins at random values of these parameters and an exhaustive search is conducted in the direction of descending gradient of *J*_*c*_. In every trial, the pendulum is balanced for 5.0 seconds and the resulting cost is recorded. The iterative tuning is terminated when the minimum cost is acquired. The selected values are *γ*_*α*_ = 0.22, *γ*_*θ*_ = 0.35, $\gamma_{\dot{\alpha }}$ = 0.15, $\gamma_{\dot{\theta }}$ = 0.18, *ω*_*α*_ = 0.88, and *ω*_*θ*_ = 0.95. Each revised function comprises of the following three terms.


$$Dissipative\;term\;\left\{ \begin{array}{l} -\rho_{\alpha }\mu_{S,\alpha }(e_{\alpha })q_{\alpha }(t)\\ -\rho_{\theta }\mu_{S,\theta }(e_{\theta })q_{\theta }(t)\\ -\rho_{\dot{\alpha }}\mu_{S,\alpha }(e_{\alpha })q_{\dot{\alpha }}(t)\\ -\rho_{\dot{\theta }}\mu_{S,\theta }(e_{\theta })q_{\dot{\theta }}(t) \end{array}\right.$$

$$Anti‐dissipative\;term\;\left\{ \begin{array}{c} \delta_{\alpha }\mu_{L,\alpha }(e_{\alpha })q_{\alpha }^{*}e_{\alpha }^{2}(t)\\ \delta_{\theta }\mu_{L,\theta }(e_{\theta })q_{\theta }^{*}e_{\theta }^{2}(t)\\ \delta_{\dot{\alpha }}\mu_{L,\alpha }(e_{\alpha })q_{\dot{\alpha }}^{*}e_{\alpha }(t){\dot{e}}_{\alpha }(t)\\ \delta_{\dot{\theta }}\mu_{L,\theta }(e_{\theta })q_{\dot{\theta }}^{*}e_{\theta }(t){\dot{e}}_{\theta }(t) \end{array}\right.$$

$$Model‐reference\;tracking\;term\;\left\{ \begin{array}{l} -\gamma_{\alpha }\mu_{M,\alpha }(e_{\alpha })(q_{\alpha }(t)-q_{\alpha }^{*})\\ -\gamma_{\theta }\mu_{M,\theta }(e_{\theta })(q_{\theta }(t)-q_{\theta }^{*})\\ -\gamma_{\dot{\alpha }}\mu_{M,\alpha }(e_{\alpha })(q_{\dot{\alpha }}(t)-q_{\dot{\alpha }}^{*})\\ -\gamma_{\dot{\theta }}\mu_{M,\theta }(e_{\theta })(q_{\dot{\theta }}(t)-q_{\dot{\theta }}^{*}) \end{array}\right.$$

The anti-dissipative terms tend to amplify the state-weighting factors to deliver a stiff control effort to alleviate large errors and disturbances and vice-versa. The dissipative term exponentially attenuates the rate-of-change in state-weighting factors during equilibrium conditions or when the anti-dissipative terms are small. The idea is to apply a softer control effort to prevent disrupted control activity, minimize the steady-state fluctuations in the state responses, and suppress the chances of plausible actuator saturation due to the anti-dissipative action. The model-reference tracking term pushes the adaptation law to generate state-weighting factors that are adequately close to the nominal weights $q_{x}^{*}$. This term contributes to reasonable performance in every condition by mimicking the nominal controller. It offers a smooth transition between dissipative and anti-dissipative action and thus prevents the controller from demonstrating undesired response in case of situations involving either large error or small error. The mild control effort offered by the model-reference tracking term helps in economizing the overall control activity as the response recovers from a transient state and finally converges to reference. Altogether, these three terms increase the controller’s adaptability to flexibly reconfigure the stiffness of damping control effort while preserving the system’s closed-loop stability. Consequently, the system acquires the capability to effectively re-modulate the damping strength and response speed of the control law against random disturbances.

**Fig 4:**
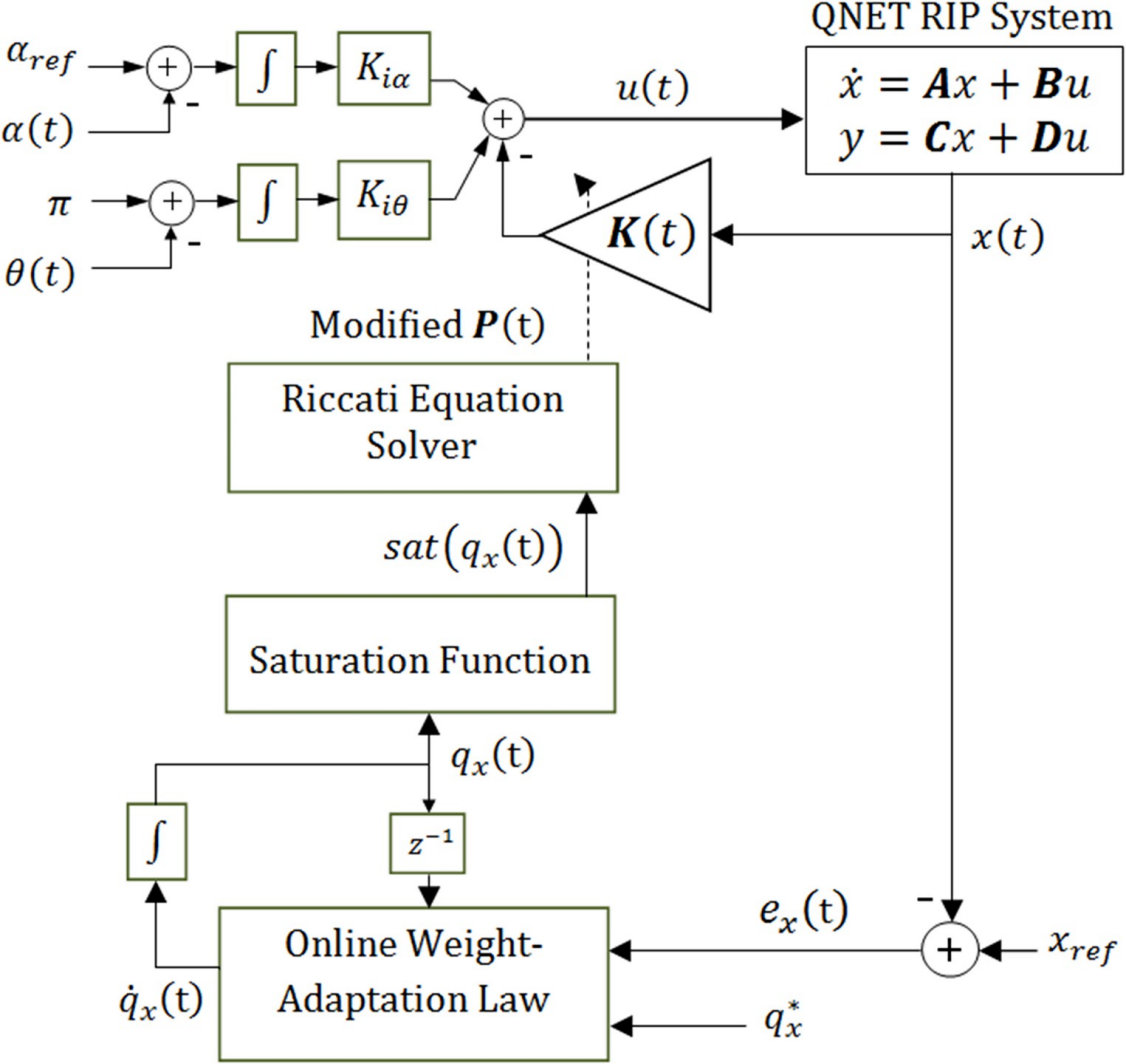
The improved variable–structure LQIC procedure.

The online adaptation starts from the nominal values of the state-weighting factors that are systematically updated online via the above-formulated algebraic functions. These differential equations are solved once after every sampling interval via numerical integration. To satisfy the LQIC’s stability requirements, the updated state-weighting factors are subjected to the saturation as shown in (23), which restricts them within ±70.0% of the nominal value. The saturated state weights are used by Riccati equation to update its solution and yield a time-varying LQIC gain vector. This control procedure is termed as Improved-Variable-Structure LQIC (IVS-LQIC) in this research. Its block diagram is illustrated in Fig 4.

## 5. Experimental evaluation and discussions

This section comprehensively discusses the experimental procedures used to emulate the real-world disturbance scenarios for the sake of analyzing each designed controller’s performance in the physical environment. To better characterize the performance of the proposed control law, the IVS-LQIC scheme is compared with the BVS-LQIC, LQIC, and the robust Sliding-Mode-Control (SMC) law proposed in [29]. The SMC scheme for this research work is implemented by using Gao’s power-rate law. It is formulated as follows [29].

$$u(t)=-{\left(F^{T}B\right)}^{-1}\left(F^{T}Ax(t)+m{\left|s(t)\right|}^{\gamma }sgn(s(t))\right)$$ (41)

where, *m* = 4.07, *γ* =0.45, and $F^{T}=[-3.16\;73.48\;-3.02\;9.25]$.

### 5.1. Experimental setup

**Fig 5:**
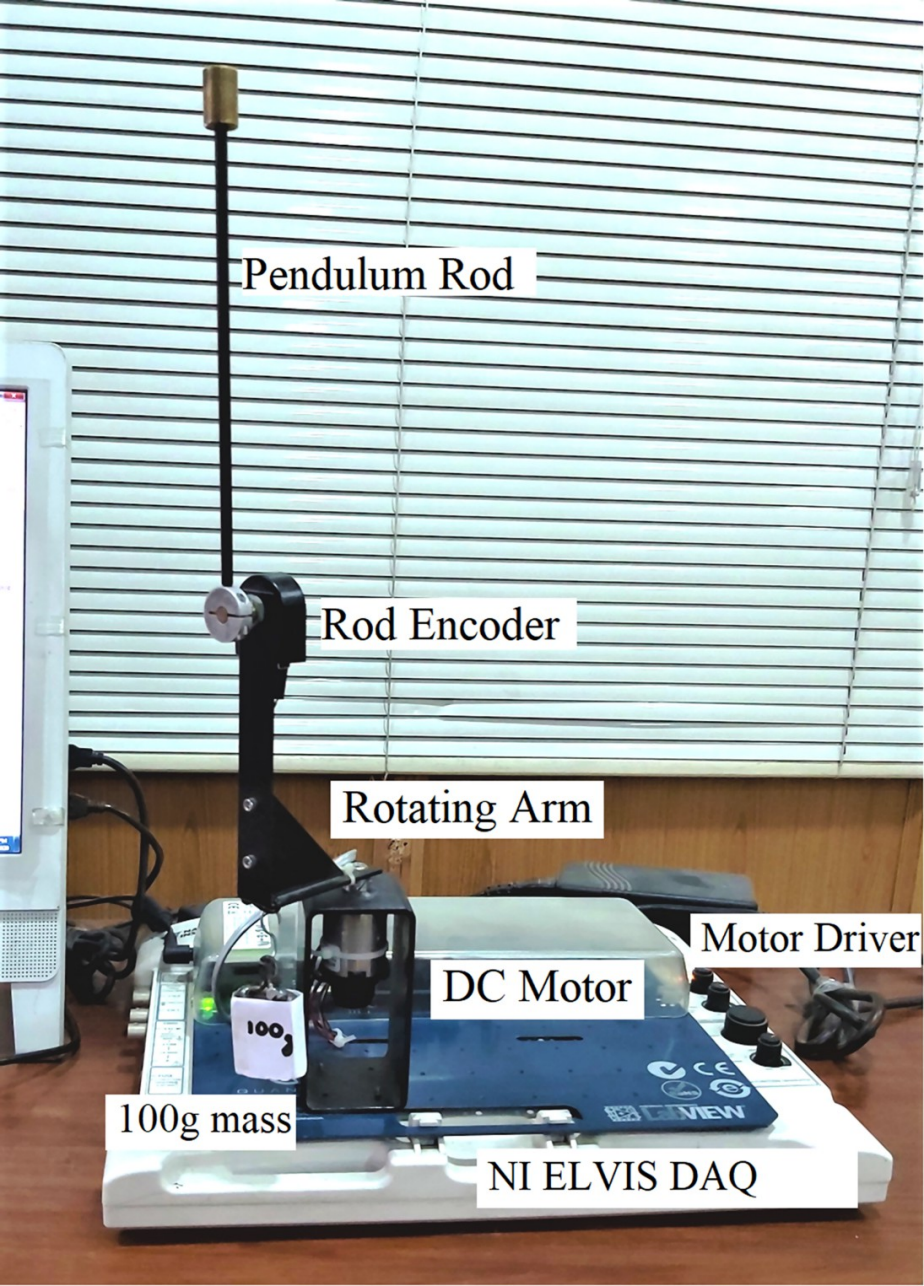
QNET Rotary Inverted Pendulum setup.

The efficacy of each control strategy is investigated in real-time using reliable hardware-in-the-loop experiments conducted on the QNET RIP setup. The snapshot of the hardware setup is illustrated in Fig 5.

**Fig 6:**
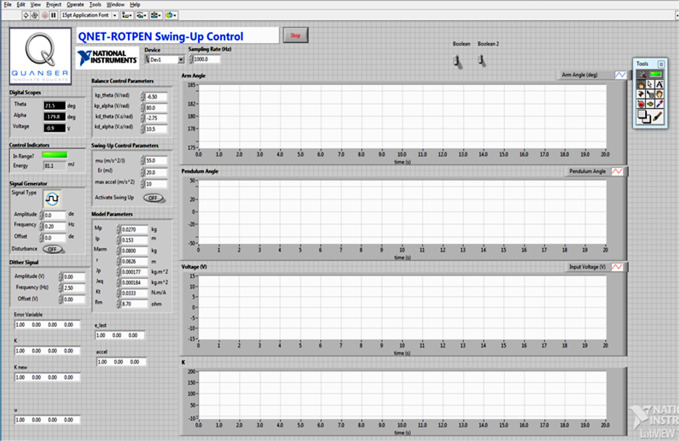
GUI of the LabVIEW control application.

The angular position of the arm and the apparatus rod is measured by the optical rotary encoders that are coupled with the motor shaft and the rod’s pivot. The angular measurements are acquired at a sampling frequency of 1.0kHz. The encoder data is filtered, digitized, and then serially fed at 9600 bps to the LabVIEW-based control software application that is running on a 64-bit, 2.1 GHz, 6.0 GB RAM embedded computer. The customized control application is implemented in the back end by using the LabVIEW’s "Block Diagram" tool. The front end of the control application acts as a Graphical-User-Interface (GUI) to display the real-time changes in *θ*(*t*), *α*(*t*), *V*_*m*_(*t*), and *K*(*t*). The GUI is shown in Fig 6.

The updated values of the system’s state error variations are used by the proposed control law to adjust the state-weighting factors, re-compute the state-compensator gains, and generate the modified control input signal. This process occurs after every sampling interval by using the embedded computer’s real-time clock. The updated modified control signals are then serially transmitted to the onboard motor driver circuit, which modulates and amplifies the control signals to actuate the motor. The motor driver is capable of safely handling the disrupted and peak control requirements of the system.

### 5.2. Tests and results

To test the robustness of the proposed control laws, each control law is tasked to maintain the pendulum rod upright while regulating the arm’s position at the desired reference, even under the influence of bounded disturbances or model variations. The performance objectives are examined using the following test cases.

#### A. Position-regulation and station-keeping

**Fig 7:**
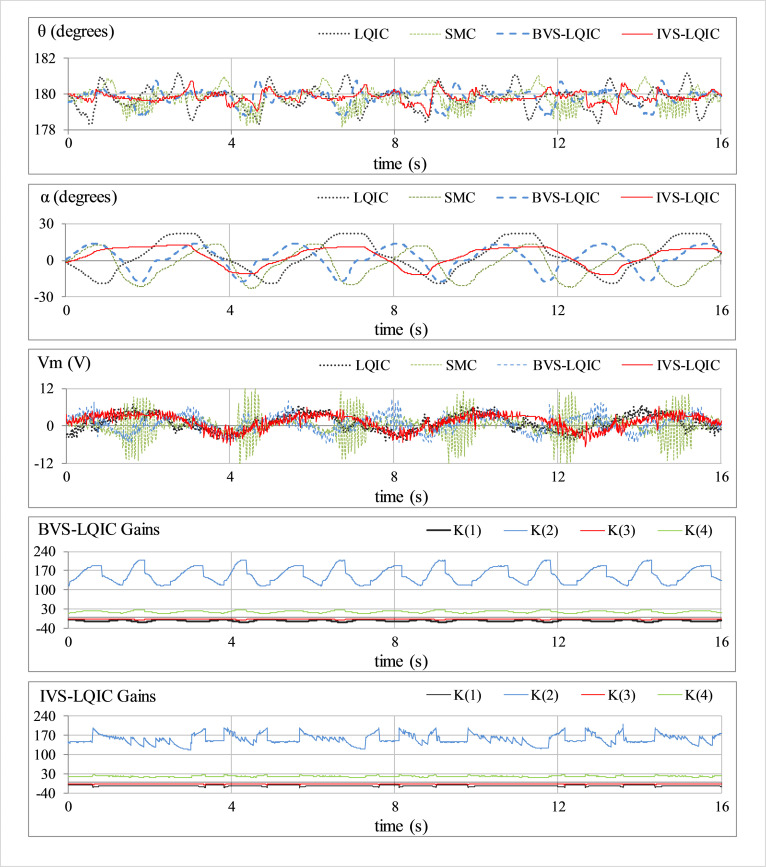
Pendulum’s response under normal conditions.

This is a preliminary test case that is used to examine the vertical position-regulation capability of the rod and the station-keeping capability of the arm. No external perturbation is applied to the hardware in this case. The corresponding variations in *θ*(*t*), *α*(*t*), *V*_*m*_(*t*), and *K*(*t*) for all the control schemes, are shown in Fig 7.

#### B. Impulsive disturbance-rejection

**Fig 8:**
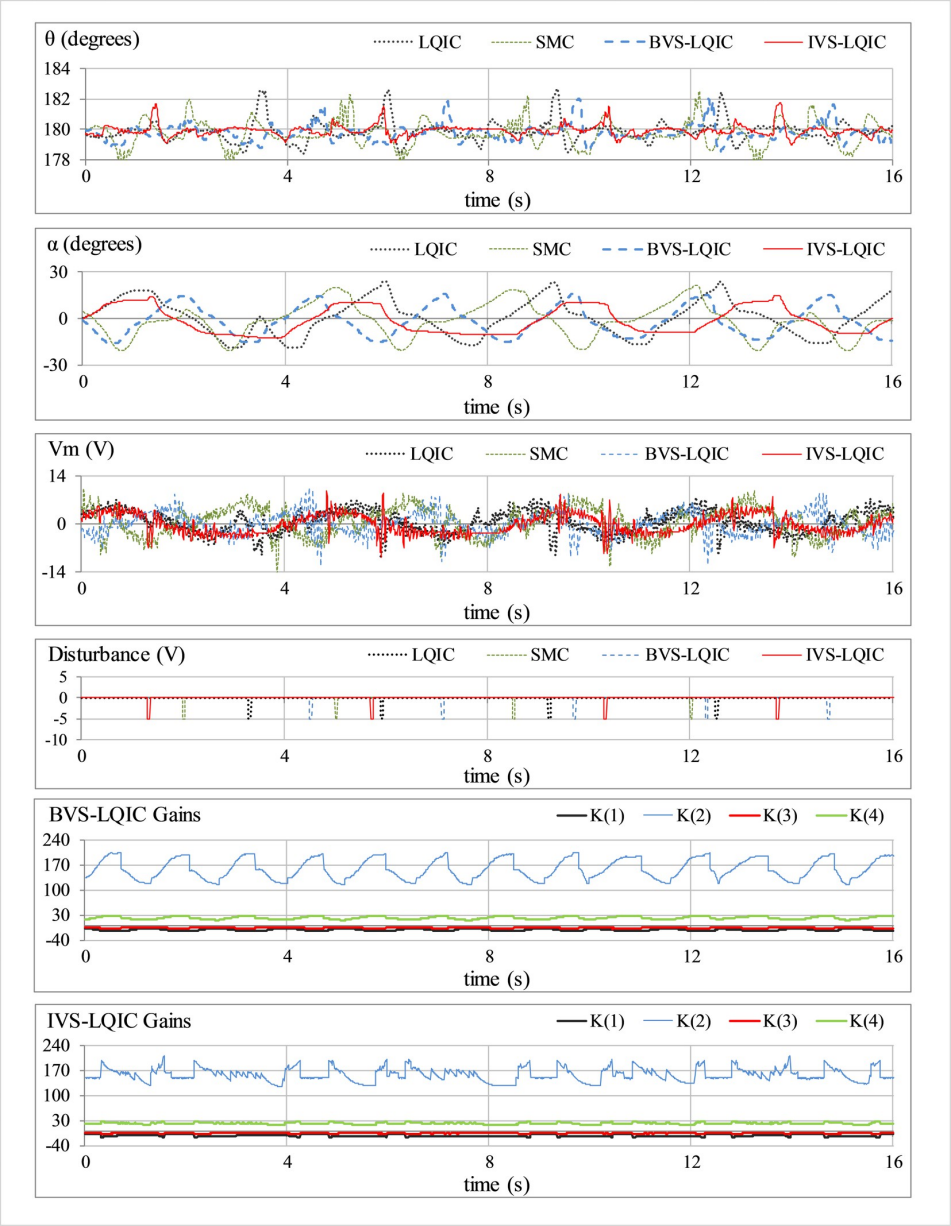
Pendulum’s response under impulsive disturbances.

The external disturbance-rejection capability of each control law is characterized by applying an impulse signal to the control input. This test case emulates the occurrence of abrupt random faults caused by environmental indeterminacies in the practical engineering systems. The response is perturbed by applying a pulse of -5.0 V and 100.0 ms duration, every time the arm approaches a local maximum. The corresponding responses of *θ*(*t*), *α*(*t*), *V*_*m*_(*t*), and *K*(*t*) for each tested control scheme, are depicted in Fig 8.

#### C. Step disturbance-rejection

**Fig 9:**
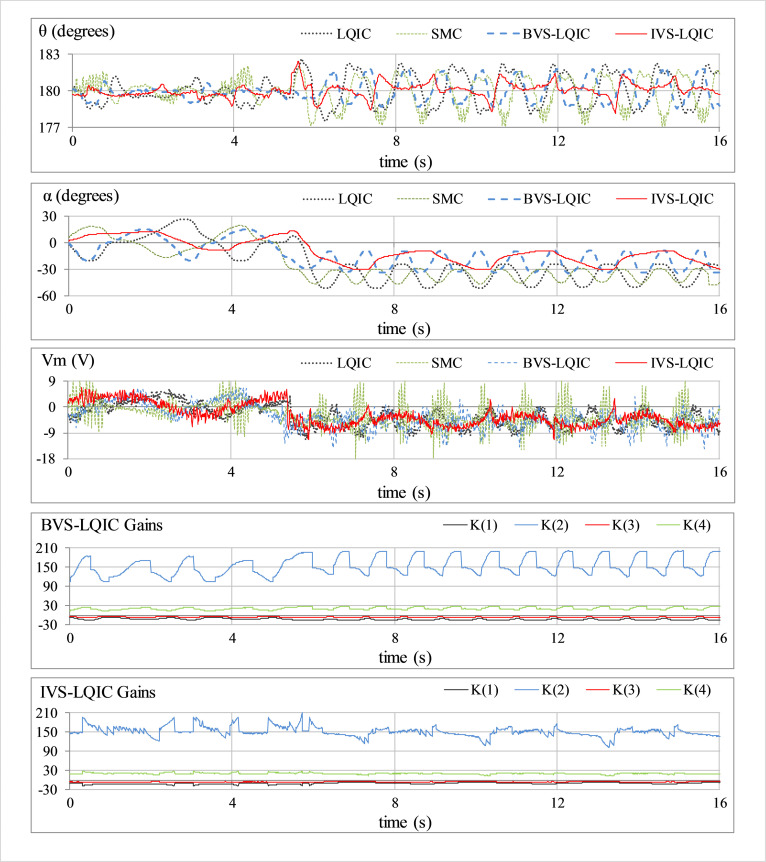
Pendulum’s response under step disturbance.

This test examines the resilience of the designed controllers against disturbances caused by external torques or abrupt but constant exogenous forces. This test case emulates the application of turbulence and wind gusts on aerospace vehicles or the application of tidal forces on marine vessels. The pendulum system is disturbed by injecting a -5.0 V step signal in the control input at t ≈ 5.0 s. The resulting responses of *θ*(t), *α*(t), *V*_*m*_(t), and *K*(t) are illustrated in Fig 9.

#### D. Noise attenuation

**Fig 10:**
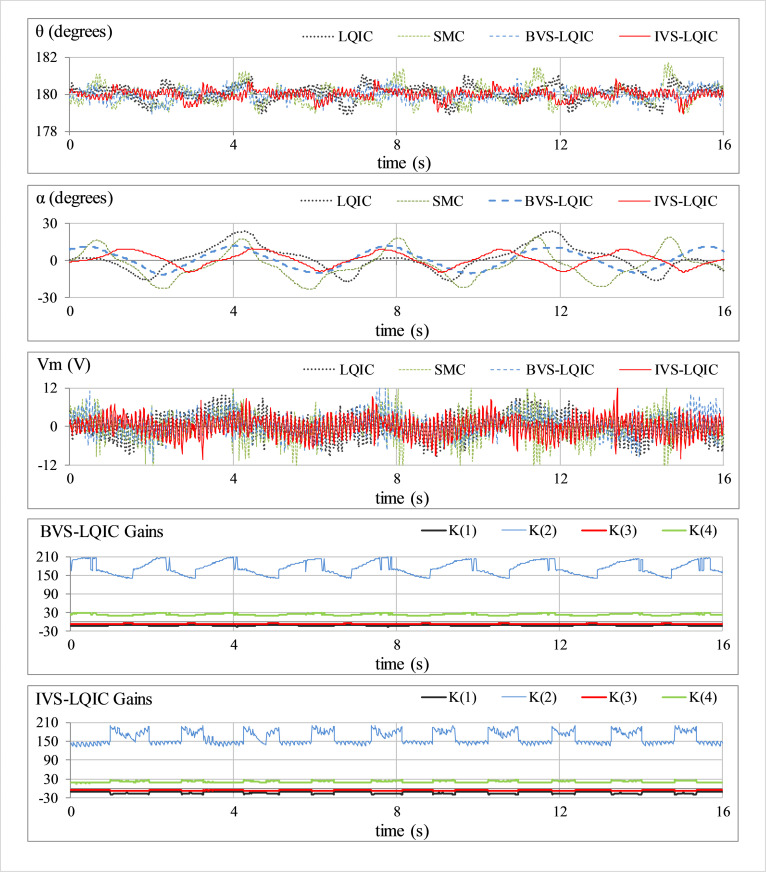
Pendulum’s response under sinusoidal disturbance.

This test case analyzes the position-regulation accuracy of pendulum’s rod and the arm under the influence of the sinusoidal disturbances. This disturbance is used to emulate the measurement noise of the sensors, mechanical vibrations, and the chattering caused by the hysteresis of the parasitic impedances in electronic circuits. The noise-attenuation capability of the pendulum is examined by applying a high-frequency signal with a low-amplitude having the form, d(t) = sin(20πt). The corresponding variations in *θ*(t), *α*(t), *V*_*m*_(t), and *K*(t) are shown in Fig 10.

#### E. Modelling-error compensation

**Fig 11:**
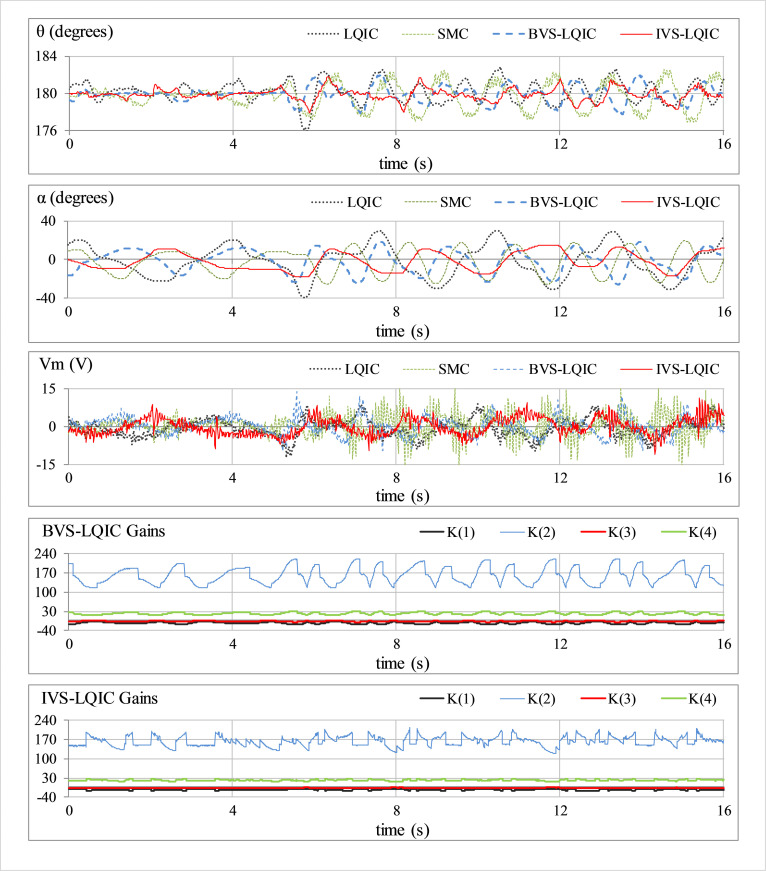
Pendulum’s response under model variation.

The insensitivity of the devised controllers against model variations or identification errors is demonstrated by attaching a mass of 0.05 kg beneath the pendulum rod as shown in Fig 5. This hardware modification introduces an error between the actual and the reference state-space models of the system. This test case emulates the occurrence of parametric uncertainties caused by un-modeled intrinsic nonlinearities in real-world engineering systems. The corresponding perturbations in *θ*(t), *α*(t), *V*_*m*_(t), and *K*(t) are shown in Fig 11.

### 5.3. Discussions

The performance of each controller, under the aforementioned test cases, is characterized by recording the following seven Critical-Performance-Indicators (CPIs).

**Table:** 

1. *e*_*x_*RMS_	: Root-mean-squared value of error in pendulum’s rod and arm.
2. MSV_m_	: Mean-squared value of motor control voltage.
3. |M_p,θ_|	: Magnitude of the peak overshoot in the rod after disturbance is applied.
4. *t*_s,*θ*_(s)	: Time taken by the rod to recover from a transient disturbance.
5. *α*_off_	: The offset in the arm’s position after disturbance is applied.
6. *α*_p-p_	: The peak-to-peak amplitude of post-disturbance oscillations in the arm.
7. V_p_	: Magnitude of peak voltage after disturbance.

The qualitative analysis of the experimental results obtained by each controller for the aforementioned tests is summarized in Table 2. A quick comparison validates the enhanced robustness of the IVS-LQIC under the influence of exogenous disturbances. The qualitative analysis of the said experimental outcomes is discussed below.

**Table 2:** Summary of experimental results.

Test	CPI	Controllers			
		LQIC	SMC	BVS-LQIC	IVS-LQIC
A	*e*_*θ_*RMS_ (degrees)	0.62	0.54	0.48	**0.35**
	*e*_*α_*RMS_ (degrees)	13.34	12.03	9.78	**8.52**
	MSV_m_ (V^2^)	8.13	11.87	7.55	**7.06**
B	*e*_*θ_*RMS_ (degrees)	0.67	0.84	0.57	**0.35**
	|M_p,θ_| (degrees)	2.70	2.52	2.01	**1.68**
	*t*_s,*θ*_(s)	0.71	0.69	0.52	**0.46**
	*e*_*α_*RMS_ (degrees)	11.44	10.78	9.50	**8.74**
	MSV_m_ (V^2^)	10.07	17.41	9.63	**7.74**
	V_p_ (V)	-10.14	-15.62	-12.33	**-9.55**
C	*e*_*θ_*RMS_ (degrees)	1.22	1.16	0.84	**0.59**
	*e*_*α_*RMS_ (degrees)	31.43	30.15	21.60	**16.62**
	*α*_off_ (degrees)	-37.02	-36.53	-22.09	**-19.08**
	*α*_p-p_ (degrees)	27.52	23.77	25.79	**20.70**
	MSV_m_ (V^2^)	24.94	26.35	23.47	**22.76**
	V_p_ (V)	-11.25	-18.01	-12.84	**-11.22**
D	*e*_*θ*_RMS_ (degrees)	0.42	0.50	0.31	**0.27**
	*e*_*α*_RMS_ (degrees)	10.14	11.78	7.55	**5.58**
	MSV_m_ (V^2^)	12.86	16.65	11.98	**9.59**
E	*e*_*θ*_RMS_ (degrees)	1.14	1.31	0.85	**0.63**
	*e*_*α*_RMS_ (degrees)	16.86	13.19	11.88	**9.56**
	MSV_m_ (V^2^)	13.17	17.76	11.72	**11.04**

In **Test A** (results shown in Fig 7), the LQIC shows a mediocre time-domain performance. The SMC exhibits relatively position-regulation behavior at the cost of large control energy expenditure. The BVS-LQIC demonstrates considerable improvement in position-regulation and control input activity as compared to LQIC. The IVS-LQIC exhibits a faster convergence rate after the initial start-up and effectively attenuates the position-regulation error throughout.

In **Test B** (results shown in Fig 8), the LQIC severely suffers from the disturbance. The SMC and BVS-LQIC systematically improve the disturbance-rejection ability, but also yield an expensive control behavior. The IVS-LQIC exhibits relatively faster transient recovery and stronger damping against overshoots (and undershoots) while minimizing the overall control energy expense by suppressing the peak servo requirements in the presence of disturbances.

In **Test C** (results shown in Fig 9), the LQIC lacks the robustness to effectively compensate for the disturbances and introduces a large offset in the arm’s position with substantial oscillations. The SMC yields robust effort to reject disturbances while contributing a highly discontinuous control activity. The BVS-LQIC exhibits reasonable improvement in disturbance compensation at the cost of a highly disrupted control activity. The IVS-LQIC offers relatively much stronger attenuation to minimize the post-disturbance offset as well as the amplitude of oscillations in the arm without requiring large actuator torques.

In **Test D** (results shown in Fig 10), the SMC demonstrates the weakest immunity against sinusoidal disturbances. The LQIC and BVS-LQIC manifest relatively better resilience against sinusoidal disturbance. The IVS-LQIC surpasses the aforesaid LQIC variants by demonstrating a drastic improvement in the noise suppression capability of the closed-loop system while relaxing constraints in terms of the control input requirements.

In **Test E** (results shown in Fig 11), the LQIC underperforms in compensating for the model variations as compared to other controllers. The SMC suppresses the perturbations in arm angle response while yielding a highly disrupted control activity. The BVS-LQIC robustly handles the model variations and rejects the post-disturbance perturbations in the responses. The IVS-LQIC delivers significantly better response speeds and damping against fluctuations and the control-input efficiency (cost) as compared to the LQIC and BVS-LQIC.

As listed in Table 2, the IVS-LQIC demonstrates better position-regulation error in the rod and arm, improved disturbance-rejection behavior, and lesser control energy expense in every test case as compared to the fixed-gain LQIC and the BVS-LQIC. The SMC demonstrates highly discontinuous control activity and relatively higher chattering content in the angular responses in every test case. The performance improvements observed in the IVS-LQIC are attributed to the enhanced self-regulating weight-adjustment law augmented with its structure, which improves the controller’s sensitivity to quickly realize the nonlinear disturbances. Moreover, it also enhances the controller’s flexibility and self-adaptability to efficiently manipulate the control profile for disturbance rejection.

## 6. Conclusion

This research formulates and experimentally validatesthe efficacy of an innovative variable structure LQIC design for the under-actuated electro-mechanical systems. The adaptive control procedure is realized by systematically constituting a self-tuning law that adapts the state-weighting factors of LQIC’s QPI in an online fashion. The self-adjusting weights tend to dynamically modify the LQIC gains, which leads to the online restructuring of the state-feedback control law after every sampling interval. The proposed adaptation law is formulated by using state-error-driven anti-dissipative terms, dissipative terms, and model-reference tacking terms. Altogether, the aforementioned three constituent terms of each weight-adjusting function increase the controller’s DOF and flexibility to yield a robust, time-optimal, and energy-efficient control effort while upholding the system’s asymptotic stability. The proposed self-tuning algorithm uses the knowledge of the past weights, the state-error variations, and model-reference tracking error in conjunction with its better self-reasoning capability to adaptively modulate the state-weighting factors online. Despite its dependence on several variables, the proposed scheme does not put an excessive computational expense on the embedded processor, and thus can be easily handled by modern digital computers. The experimental outcomes justify the aforementioned claims by yielding faster transient recovery behavior and strong damping effort to reject the nonlinear disturbances while preserving the system’s closed-loop stability and relaxing constraints in terms of the control input requirements of the actuator. In the future, the proposed adaptation mechanism can be extended and applied to other nonlinear complex dynamical systems. Moreover, the flexibility, control yield, and computational complexity of state-of-the-art soft-computing techniques (such as fuzzy systems and artificial neural networks) can also be investigated and compared with the proposed weight adaptation scheme for similar control applications.

## References

[1] Mahmoud MS. Advanced Control Design with Application to Electromechanical Systems. 1st ed. Netherlands: Elsevier Science; 2018.

[2] Li Z, Yang C, Fan L. Advanced Control of Wheeled Inverted Pendulum Systems. London: Springer, 2013.

[3] Gritli H, Belghit S. Robust feedback control of the underactuated Inertia Wheel Inverted Pendulum under parametric uncertainties and subject to external disturbances: LMI formulation. J Franklin Inst. 2018; 355(18): 9150–9191.

[4] An-chyau H, Yung-feng C, Chen-yu K. Adaptive Control of Underactuated Mechanical Systems. Singapore: World Scientific; 2015.

[5] Szuster M, Hendzel Z. Intelligent Optimal Adaptive Control for Mechatronic Systems. Cham: Springer; 2017.

[6] Ahmad E, Iqbal J, Arshad M, Liang W, Youn I. Predictive control using active aerodynamic surfaces to improve ride quality of a vehicle. Electronics, 2020, 9(9):1463.

[7] Khan O, Pervaiz M, Ahmad E. Iqbal J. On the derivation of novel model and sophisticated control of flexible joint manipulator. Revue Roumaine des Sciences Techniques-Serie Electrotechnique et Energetique, 2017, 62(1): 103–108.

[8] Anjum M, Khan Q, Ullah S, Hafeez G, Fida A, Iqbal J, et al. Maximum power extraction from a standalone photo voltaic system via neuro-adaptive arbitrary order sliding mode control strategy with high gain differentiation. Applied Sciences, 2022, 12(6): 2773

[9] Tang Y, Zhou D, Jiang W. A New Fuzzy-Evidential Controller for Stabilization of the Planar Inverted Pendulum System. PLoS ONE. 2016; 11(8): 1–16. doi: 10.1371/journal.pone.0160416 27482707 PMC4970802

[10] Asghar A, Iqbal M, Khaliq A, Rehman S, Iqbal J. Performance comparison of structured H∞ based looptune and LQR for a 4-DOF robotic manipulator. PLoS ONE 2022; 17(4): e0266728. 35404940 10.1371/journal.pone.0266728 PMC9000114

[11] Ajwad SA, Iqbal J, Islam RU, Alsheikhy A, Almeshal A, Mehmood A. Optimal and robust control of multi DOF robotic manipulator: Design and hardware realization. Cybernet Syst 2018; 49(1): 77–93.

[12] Saleem O, Mahmood-ul-Hasan K, Rizwan M. An experimental comparison of different hierarchical self-tuning regulatory control procedures for under-actuated mechatronic systems. PLoS ONE. 2021; 16(8): 1–35. doi: 10.1371/journal.pone.0256750 34460842 PMC8405034

[13] Ahmadi A, Mohammadi-Ivatloo B, Anvari-Moghaddam A, Marzband M. Optimal Robust LQI Controller Design for Z-Source Inverters. Applied Sciences. 2020; 10(20):7260.

[14] Calgan Haris, Demirtas Metin, A robust LQR-FOPIλDμ controller design for output voltage regulation of stand-alone self-excited induction generator. Electric Power Systems Research, Volume 196, July 2021, 107175

[15] Smith AMC, Yang C, Ma H, Culverhouse P, Cangelosi A, Burdet E. Novel Hybrid Adaptive Controller for Manipulation in Complex Perturbation Environments. PLoS ONE. 2015; 10(6): 1–19. doi: 10.1371/journal.pone.0129281 26029916 PMC4452518

[16] Batmani Y, Davoodi M, Meskin N. Nonlinear Suboptimal Tracking Controller Design Using State-Dependent Riccati Equation Technique. IEEE Trans Control Syst Technol. 2017; 25(5): 1833–1839.

[17] Zhang H, Wang J, Lu G. Self-organizing fuzzy optimal control for under-actuated systems. J Syst Control Eng. 2014; 228(8): 578–590.

[18] Saleem O, Mahmood-ul-Hasan K. Hierarchical adaptive control of self-stabilizing electromechanical systems using artificial-immune self-tuning mechanism for state weighting-factors. J Mech Sci Technol. 2021; 35, 1235–1250.

[19] Saleem O, Mahmood-Ul-Hasan K. Indirect Adaptive State-Feedback Control of Rotary Inverted Pendulum Using Self-Mutating Hyperbolic-Functions for Online Cost Variation. IEEE Access. 2020; 8(1): 91236–91247.

[20] Saleem O, Mahmood-ul-Hasan K. Robust stabilisation of rotary inverted pendulum using intelligently optimised nonlinear self-adaptive dual fractional order PD controllers. Int J Syst Sci. 2019; 50(7): 1399–1414.

[21] Jian Z, Yongpeng Z. Optimal Linear Modeling and its Applications on Swing-up and Stabilization Control for Rotary Inverted Pendulum. In: Proceedings of the 30th Chinese Control Conference. 2011; Yantai, China: pp. 493–500.

[22] Balamurugan S, Venkatesh P. Fuzzy sliding-mode control with low pass filter to reduce chattering effect: an experimental validation on Quanser SRIP. Sadhana. 2017; 42: 1693–1703.

[23] Astom KJ, Apkarian J, Karam P, Levis M, Falcon J. Student Workbook: QNET Rotary Inverted Pendulum Trainer for NI ELVIS. Ontario: Quanser Inc.; (2011).

[24] Lewis FL, Vrabie D, Syrmos VL. Optimal Control. New Jersey: John Wiley and Sons; 2012.

[25] Das S, Pan I, Halder K, Das S, Gupta A. LQR based improved discrete PID controller design via optimum selection of weighting matrices using fractional order integral performance index. Appl Math Model. 2013; 37(6): 4253–4268.

[26] Saleem O, Omer U. Synergistic Speed Control Strategy for PMDC Motor. In: Proceedings of IEEE 20th International Multitopic Conference (INMIC’ 17). 2017; Lahore, Pakistan: pp. 1–6.

[27] Fisher AD, VanZwieten JH Jr., VanZwieten TS. Adaptive Control of Small Outboard-Powered Boats for Survey Applications. In: OCEANS’09 Proceedings of the MTS/IEEE Marine Technology for Our Future: Global and Local Challenges. 2009; Biloxi, Mississippi: pp. 1–9.

[28] Balestrino A, Caiti A, Calabr V, Crisostomi E, Landi A. Chaper 5—From Basic to Advanced PI Controllers: A Complexity vs. Performance Comparison. In: Yurkevich VD, editors, Advances in PID Control. Croatia: Intech; 2011. pp. 85–100.

[29] Saleem O, Mahmood-ul-Hasan K. Adaptive State-space Control of Under-actuated Systems Using Error-magnitude Dependent Self-tuning of Cost Weighting-factors. Int J Control Autom Syst. 2021; 19(02): 931–941.

